# An Overview of Meta-Analyses of Endovascular Bridging Therapies for Acute Ischemic Stroke

**DOI:** 10.1155/2018/9831210

**Published:** 2018-03-07

**Authors:** Si-Yan Chen, Xing-Ru Zhang, Jie Chen, Wan-Qian Ge, Wen-Wen Wang, Xin-Shi Wang, Cheng-Long Xie

**Affiliations:** ^1^Department of Neurology, The First Affiliated Hospital of Wenzhou Medical University, Wenzhou 325000, China; ^2^The Center of Traditional Chinese Medicine, The Second Affiliated Hospital and Yuying Children's Hospital of Wenzhou Medical University, Wenzhou 325027, China

## Abstract

**Background:**

Acute Ischemic Stroke (AIS) is a common cause of death worldwide and the leading cause of long-term severe disability. Endovascular bridging therapies (EBT), including endovascular thrombectomy (ET) and intra-arterial thrombolytic (IAT), have been recommended to realize a favorable functional outcome for AIS patients.

**Methods:**

An overview of meta-analyses of primary randomized controlled trial (RCT) studies was performed evaluating EBT for AIS patients compared with usual care.

**Results:**

Ten meta-analyses were included in this overview. ET was associated with a higher incidence of achieving functional outcome improvement, defined as a modified Rankin scale of 0 to 1 (mRS, *p* = 0.003), 0 to 2 (*p* < 0.00001), and 0 to 3 (*p* = 0.005). The risk of symptomatic intracranial hemorrhage (sICH) rate and all-cause mortality were similar between the two groups. Moreover, IAT treatment was also related to significantly improved outcomes in terms of the mRS score (*p* < 0.05), but no significant difference in rates of sICH and mortality within 90 days.

**Conclusions:**

In conclusion, our analysis supports that EBT, regardless of format (e.g., ET or IAT), is superior to the best medical therapy alone (e.g., IVT) in terms of mRS score in patients with AIS. In addition, the safety of EBT is similar to IVT.

## 1. Introduction

Stroke is a common cause of death worldwide and the leading cause of long-term severe disability. Over 80% of all incident strokes are ischemic, resulting from an occluding thrombus of a cerebral artery [1]. Intravenous recombinant tissue plasminogen activator (rt-PA) administered as early as possible within 4.5 hours is recommended after the onset of acute ischemic stroke (AIS) which improves survival rate and functional outcomes [2] and is the most proven helpful therapy for the emergency management of AIS [3]. Nevertheless, intravenous rt-PA (IVT) has multiple limitations, containing nonresponsiveness of large thrombus to rapid dissolution, a narrow time window for treatment [4], the risk of cerebral and various systems hemorrhage, and several important contraindications, such as recent surgery, coagulation abnormalities, or head injury within the past 3 months [5]. Consequently, as few as 10% of AIS patients can be qualified for administration with IVT [6]. Furthermore, some AIS patients with intracranial obstruction of internal carotid artery (ICA) or the middle cerebral artery (MCA) are associated with long reperfusion times and poor revascularization rates, IVT results in early recanalization approximate 10 to 50% [7], and the prognosis of these patients remains poor, which lead to exploration of new endovascular bridging therapy for AIS patients [8].

Endovascular bridging therapies (EBT) include intra-arterial pharmacologic thrombolysis (IAT) or manipulation of the clot with the use of stent-retriever technology after IVT. EBT are given through the large arteries more frequently and rapidly than IVT in patients with AIS and are utilized to cure the patients with occlusions of the large intracranial arteries [9]. Endovascular treatment of AIS with IAT has been revealed as a vital therapeutic modality [10] and conferred a markedly greater chance of realizing favorable functional outcomes compared with IVT for AIS patients [11]. Meanwhile, endovascular thrombectomy (ET) with mechanical devices is a straight reperfusion way that is different from pharmacologic thrombolysis [12]. Endovascular mechanical treatments can recanalize large arterial occlusions and remove proximal clots rapidly and lead to higher rates of reperfusion than IVT alone [13]. At this stage, it is significant to establish the efficacy and safety of EBT in patients with AIS and furthermore to address the pivotal factors that determine their success or failure. Until now, several systematic reviews or meta-analyses have been conducted to assess EBT in treatment of AIS. We will comment on this evidence in light of the overall effectiveness of EBT interventions in the separate systematic reviews. By doing so, to bring together all relevant published information based on the large amount of available data, we here offer an overview of those published systematic reviews.

## 2. Materials and Methods

We implemented this overview of meta-analyses based on the modified Preferred Reporting Items for Systematic Reviews and Meta-Analyses (PRISMA) statement (see the additional file: PRISMA checklist (available here)).

### 2.1. Search Strategy

Systematic literature searches of PubMed, Cochrane Library, and Google Scholar databases published from their inception to January 2017 were performed to identify studies addressing the role of EBT (containing intra-arterial thrombolysis and endovascular thrombectomy after intravenous rt-PA) in AIS patients management. The search terms used were various relevant combinations: “endovascular or intra-arterial or fibrinolysis or thrombolysis or thrombectomy” AND “ischemia or stroke” AND “systematic review or meta-analysis”. Reference lists of studies with potential all relevant articles were manually screened for additional studies that were not previously identified.

### 2.2. Inclusion and Exclusion Criteria

For this overview of reviews, we included only systematic reviews or meta-analyses of RCTs (randomized controlled trials) of EBT in participants with AIS. The included systematic reviews had to live up to the following criteria.

*Participants.* Adults (aged 18 years and over) who reported to suffer acute ischemic stroke within 12 hours were included.

*Interventions and Comparison*. Meta-analyses of RCTs assessing “intra-arterial administration of thrombolytic drugs (e.g., IAT) or the use of various thrombectomy devices (e.g., ET)” after IVT as the intervention compared with IVT alone were considered. IAT was known as chemical dissolution of blood clots with locally delivered thrombolytic; ET was defined as the intra-arterial utilization of a micropipe or other devices for mechanical thrombectomy.

*Primary Outcomes and Secondary Outcomes.* The primary outcome measure is the functional index using the modified Rankin scale (mRS). This 7-score ordinal scale ranges from 0 (no symptoms) to 6 (death). Secondary outcomes studied were symptomatic intracranial hemorrhage (sICH), failure to recanalize, and mortality.

### 2.3. Data Extraction and Analysis

All systematic reviews were read by two independent authors (WQG, JC) and data from the studies were extracted using a Microsoft Excel spreadsheet and validated according to predefined criteria. Original, two authors (WQG, JC) received a detailed introduction to the overview protocol and then independently screened a small sample of records to pilot test screening forms. In case data from the systematic reviews were unclear or seemed to be missing, we touched the correspondence authors for statement and found original RCTs for details. Any disagreements or disputes were resolved through discussion, and a third overview author (CLX) acted as a referee if necessary. We extracted and summarized the following details from the included article: author's name, the publication year, number of included studies and participants, type of intervention, comparator, average time from onset to treatment, length of follow-up, outcome measures, and the conclusions of the included meta-analyses.

### 2.4. Quality Assessment of Included Reviews

Eligible systematic reviews were quality assessed using AMSTAR questionnaire, an instrument used to evaluate the methodological quality of systematic reviews [14] and the degree to which reviews are biased by comparing them on the basis of distinct criteria. Eleven AMSTAR items are as follows: (1) Was a priori design provided? (2) Was a comprehensive and detailed literature search performed? (3) Was there duplicate study selection and data extraction? (4) Was the status of publication used as an inclusion criterion? (5) Was a list of included and excluded studies provided? (6) Were the basic characteristics of included studies provided? (7) Was the scientific quality of the included studies assessed and documented? (8) Was the scientific quality of the included studies used appropriately in formulating conclusions? (9) Were the methods used to combine the findings of studies appropriate? (10) Was the likelihood of publication bias assessed? (11) Was the conflict of interest stated? Ratings used in AMSTAR include “yes” (clearly done), “no” (clearly not done), and “not applicable.”

## 3. Results

### 3.1. Description of the Screening Process

**Figure 1:**
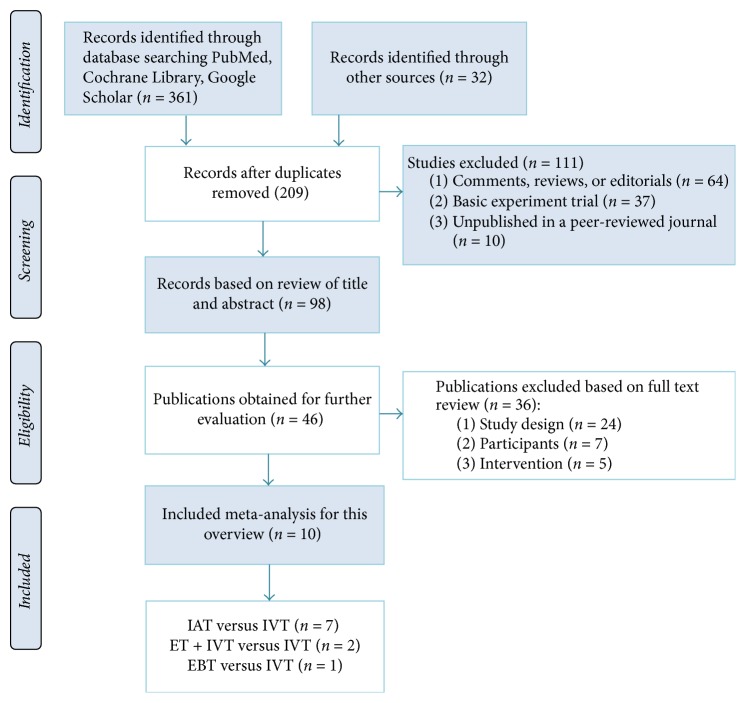
Study flow diagram.

The searches identified potential meta-analyses and the total number of papers found in three database was 361. After eliminating duplicate papers, 209 papers remained. Through screening the titles and abstracts, we further ruled out 111 papers in this stage. In the remaining 98 papers, after checking the full texts, finally 10 meta-analyses were included in this overview [15–24]. Among them, seven meta-analyses focused on the use of ET after IVT as the intervention compared with IVT alone, whilst two studies compared IAT + IVT with IVT, and the remaining one study compared EBT with IVT. The detail of search process is shown in Figure 1.

### 3.2. Study Characteristics and Assessing the Quality of Meta-Analysis

We selected the most recent updates of meta-analyses of thrombectomy devices (ET) or intra-arterial administration of thrombolytic drugs (IAT) for treating AIS compared with IVT for inclusion in this overview. Relevant primary studies in the included 10 meta-analyses were demonstrated in Table 1. We found that meta-analyses included between 3 and 9 primary studies meeting the inclusion criteria and some studies were included in multiple meta-analyses, especially five studies of MR CLEAN, ESCAPE, EXTEND-IA, SWIFT PRIME, and REVASCAT. The basic characteristics and major conclusions of included meta-analyses are shown in Table 2. The 10 meta-analyses included were published between 2010 and 2016. Following quality assessment using AMSTAR, nearly all eligible meta-analyses were deemed high quality and included for full review. All meta-analyses reported mRS score at 3 months as a primary outcome and nine studies reported symptomatic intracranial hemorrhage and mortality as a secondary outcome. Nevertheless, only two studies showed the data about revascularization at 24 hours. In this overview, no meta-analysis achieved a perfect score of 11/11, though six achieved 10/11, three scored 9/11, and the remaining one got 8/11. Quality assessment results are displayed in Table 2. Criteria which scored badly using the AMSTAR tool were “provide a priori design (question 1)” and “the status of publication used as an inclusion criterion (question 4).”

### 3.3. ET + IVT versus IVT

**Figure 2:**
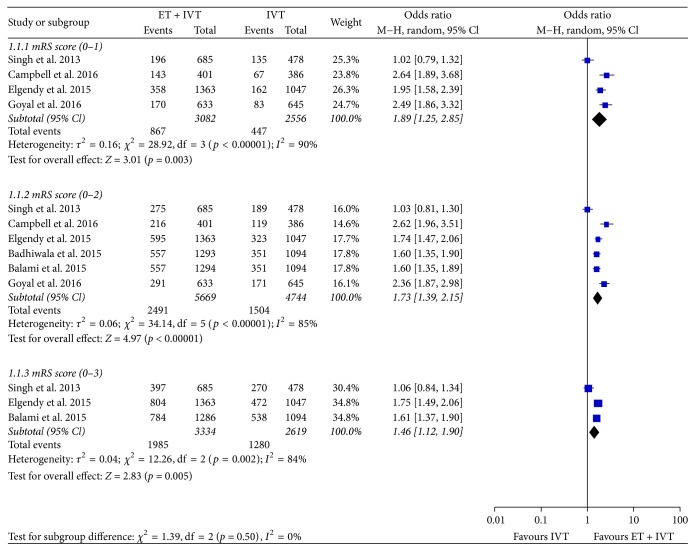
Meta-analysis of EBT versus standard therapy for the outcome of proportional treatment benefit across mRS scores (0 to 1, 0 to 2, and 0 to 3, resp.) at 90 days. EBT: endovascular bridging therapies; mRS: modified Rankin scale.

Pooling the result of mRS score was more favorable with ET relative to standard therapy, with greater proportions of AIS patients in each category of favorable outcome (mRS 0-1, 0–2, or 0–3, resp.). For the primary outcome of mRS score reduction, four studies [18–21] consistently showed reduced chance of disability at 3 months in patients assigned to ET versus those assigned to control (*p* < 0.05, Table 3). In terms of mRS score (0-1), ET was associated with significantly higher rates of functional independence at 3 months (OR 1.89; 95% CI: 1.25–2.85; *p* = 0.003; Figure 2); mRS score (0–2) was available from 6 trials of seven; then we pooled the whole data and found significant difference in favor of ET compared to IVT (OR 1.73; 95% CI: 1.39–2.15; *p* < 0.00001; Figure 2); meanwhile, a statistical significant effect was obtained between ET and IVT based on the mRS score 0–3 (OR 1.46; 95% CI: 1.12–1.90; *p* = 0.005; Figure 2).

Secondary outcomes of mortality and sICH rate at 3 months among both groups for all trials are detailed in Table 4. There were six meta-analyses that mentioned mortality and sICH rate and the results were not difference at all. Singh et al. [15] showed that ET is not superior to IVT in improving mortality (17.9% versus 17.1%, *p* > 0.05) with a similar rate of symptomatic hemorrhage (5.9% versus 6.1%, *p* > 0.05). One study conducted by Balami et al. [16] demonstrated that no significant difference was found in the prespecified secondary outcomes mortality (OR 0.84; 95% CI: 0.64–1.05; *p* = 0.12) and sICH (OR 1.03; 95% CI: 0.71–1.49; *p* = 0.88) when ET was compared with IVT. However, Elgendy et al. [17] displayed that ET after usual care was associated with a trend toward reduction in the risk of all-cause mortality (15.9% versus 17.9%, *p* = 0.82) and sICH rate (5.1% versus 5.0%, *p* = 1.02) compared with usual care alone, but still not significant. Similarly, mortality at 90 days and risk of sICH rate did not differ between populations in the remaining three meta-analysis (mortality: *p* = 0.27, *p* = 0.16, and *p* = 0.12, resp.; sICH: *p* = 0.56, *p* = 0.8, and *p* = 0.76, resp.). In addition, the results of revascularization at 24 hours were conclusive in the two meta-analyses. Elgendy et al. [17] reported that ET after usual care was related to improved recanalization compared with usual care alone (66.6% versus 39.2%, *p* = 0.0001). Meanwhile, one meta by Badhiwala et al. [18] showed that rates of angiographic revascularization at 24 hours for ET were 75.8% versus 34.1% for standard therapy (OR 6.49; 95% CI: 4.79–8.79; *p* < 0.01). Efficacy of secondary outcomes from the pooled data is shown in Table 4.

### 3.4. IAT + IVT versus IVT

We identified two meta-analyses comparing IAT with IVT in AIS patients. Pooling the results conducted by Lee et al. [22] reported that IAT was associated with increased good clinical outcomes according to mRS score 0–2 (OR 2.05; 95% CI: 1.33–3.14; *p* = 0.001) and excellent clinical outcomes based on the mRS score 0-1 (OR 2.14; 95% CI: 1.31–3.51; *p* = 0.003). For additional end points, IAT also increased frequencies of minimal neurologic deficit and reduced impairment of activities of daily living and partial or complete recanalization. Fields et al. [23] indicated that IAT treated patients were significantly more likely to have a mRS 0-1 (31% versus 20%, *p* = 0.01); mRS 0–2 (43% versus 31%, *p* = 0.01); and NIHSS score (*p* = 0.007) at 3-month follow-up. Table 3 showed the detailed data of the included meta-analyses. In terms of safety question, both meta-analyses reported that IAT was associated with increased sICH rate (8.9% versus 2.3%, *p* = 0.02; 11% versus 2%, *p* = 0.02, resp.). However, there was no difference in mortality between IAT and IVT groups (20.5% versus 24.0%, *p* = 0.46; 20.0% versus 19.0%, *p* = 0.57, resp.). Consequently, increased sICH frequencies are not associated with any increase in mortality based on these two meta-analyses.

### 3.5. EBT versus IVT

Only one meta-analysis comparing EBT with IVT in AIS patients indicated that the general effectiveness of EBT was much better than IVT. Fargen et al. [24] reported that primary outcome (mRS 0–2 at 3 months) occurred significantly more frequent in patients randomized to EBT compared with IVT (39.1% versus 32.6%, *p* = 0.018, Table 3). Conversely, the secondary outcome of mRS 0-1 did not show significant discrepancy between two groups (*p* = 0.09). Finally, mRS 0–3 at 90 days occurred markedly more continually in the EBT arm (*p* = 0.019) and the mortality did not show obvious difference between the two groups (*p* = 0.73).

## 4. Discussion

### 4.1. Summary of Evidence

Ten meta-analyses were included in the present overview. To our knowledge, this is the first overview for meta-analyses on efficacy and safety of EBT for AIS patients. We found that EBT, regardless of format (e.g., ET or IAT), is superior to the best medical therapy alone (e.g., IVT) in terms of mRS score in patients with acute ischemic stroke. Compared with control groups, adverse events were similar in patients treated with EBT compared with IVT regarding the symptoms of intracranial hemorrhage and mortality risk did not apparently differ between groups.

### 4.2. Thrombectomy

The conclusions that can be drawn about the efficacy of thrombectomy for AIS are solid because they are based on seven meta-analyses and several large multicenter case registries. One special advantage of our meta-analysis is that it contains only trials that incorporated crucial elements of current clinical practice, such as mechanical thrombectomy after usual care (e.g., intravenous alteplase) and universal requirement for proven large artery occlusion and timely treatment (within 6 h). Meanwhile, three meta-analyses [19–21] utilized the second-generation stent retrievers and demonstrated robust benefit of such mechanical device for AIS patients, approaching a double increase in odds of achieving a beneficial shift in mRS score. Goyal et al. [19] and Campbell et al. [21] reported that the extent of benefit conferred by ET is substantial; for every 100 patients treated, approximately 40 will have a less disabled outcome than with IVT, and nearly 23 more will achieve an independent outcome (mRS 0–2) as a result of treatment. Overall, all meta-analyses included in this overview showed a trend for better outcomes with ET. Moreover, advanced and detailed imaging before ET leads to a delay in the time to revascularization. Elgendy et al. [17] demonstrated that advanced imaging has not been shown to affect patient outcomes or increase the risk of sICH rate, but it helps to select appropriate patients for ET.

### 4.3. Intra-Arterial Thrombolysis

Only two meta-analyses [22, 23] mentioned IAT treatment after IVT as the intervention compared with IVT alone for AIS patients and the results were significant. In one meta-analysis [23], the data pooled from three studies provide a precise estimate of the profitable effect of IAT when administered within 6 hours of symptom occurrence for the therapy of AIS due to middle cerebral artery (MCA) occlusion. Although a total of just 334 AIS patients were enrolled in these three studies and the number of studies and patients was limited, a clinically and statistically robust increase in the probability of a favorable functional outcome mainly according to mRS score was observed in the IAT compared with control subjects. Three primary studies included in this meta-analysis have similar time window (<6 h) and artery occluded (M1 segment of the MCA); all of these factors contribute to the homogeneous results and are easy to generalize. Moreover, although this overall benefit came at the cost of increased risk of sICH rate, with an OR of 4.6 in the IAT treated group, there were no influence and discrepancy on 90-day mortality. Another meta-analysis conducted by Lee et al. 2010 [22] based on five studies showed that beneficial effects of IAT after IVT were highly statistically difference for increased rates of both good (14.8%) and excellent (13.0%) clinical outcomes. The chances of these outcome measures were more than doubled compared to control groups.

### 4.4. Adverse Effects

All the treatments considered in this overview carry the risk of side-effects (mortality and sICH rate). There were nine meta-analyses [15–19, 21–24] that mentioned mortality and the results were not difference at all (Table 4). In terms of sICH rate, the risk of in-hospital sICH was similar between ET and IVT groups based on six meta-analyses. Nonetheless, two meta-analyses [22, 23] of IAT compared with IVT showed increased sICH frequencies; however, the results are not associated with any increase in mortality. The sample size of included studies in this review was enough for quantification of serious adverse events and found no difference between two arms. In a word, the safety of EBT is similar to IVT in clinic. The results of such meta-analyses could show the development of clinical practice guidelines. In addition, it may be beneficial for medical personnel involved in the early care of patients with AIS and using ET (after IVT) as the first choice when encountering candidate patients compared with IVT alone.

### 4.5. Limitations

There are some limitations to this overview. First, the included meta-analyses were heterogeneous and were not necessarily comparable. They were carried out on different regions, with different enrollment criteria, and used variable endovascular therapies tools. Second, all of the included meta-analyses specified that AIS patients were included on the basis of CT imaging, and EBT was conducted quickly once imaging eligibility had been ascertained, whereas the benefit of EBT was not analysis alone for some of patients with poor collateral grade or unfavorable penumbral patterns in this paper. The capacity to provide adjusted treatment effect for some subgroups analysis was limited. Third, three included meta-analyses [19–21] focused on the endovascular trials using the Solitaire device and the remaining four utilized other endovascular devices or Solitaire mix. The direct comparison was missing and unable to draw the definite conclusions which was better. However, several studies confirmed the robust treatment benefits when using the Solitaire device in AIS patients with large vessel occlusion ischemic stroke.

## 5. Conclusions

In conclusion, our analysis supports that EBT, regardless of format (e.g., ET or IAT), is superior to the best medical therapy alone (e.g., IVT) in terms of mRS score in patients with AIS. In addition, the safety of EBT is similar to IVT.

## Figures and Tables

**Table 1:** Relevant primary studies in the included meta-analysis (RCTs).

	ET + IVT versus IVT							IAT versus IVT		EBT versus IVT
Primary studies from meta-analysis	Singh et al. 2013	Balami et al. 2015	Elgendy et al. 2015	Badhiwala et al. 2015	Goyal et al. 2016	Bush et al. 2016	Campbell et al. 2016	Lee et al. 2010	Fields et al. 2011	Fargen et al. 2015
First author (year)	*N* = 5	*N* = 8	*N* = 9	*N* = 8	*N* = 5	*N* = 5	*N* = 4	*N* = 5	*N* = 3	*N* = 6
PROACT (1998)								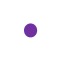	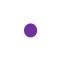	
PROACT II (1999)								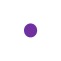	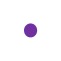	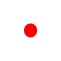
Keris et al. (2001)								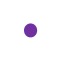		
Macleod et al. (2005)								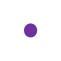		
MELT (2007)								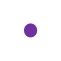	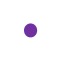	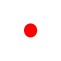
Sen et al. (2009)	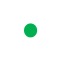									
Ciccone et al. (2010)	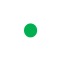									
SYNTHESIS (2013)	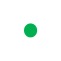	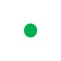		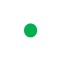						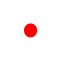
MR RESCUE (2013)	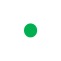	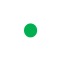	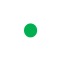	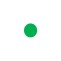						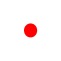
IMS III (2013)	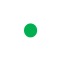	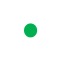	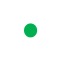	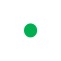						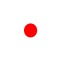
MR CLEAN (2015)		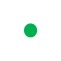	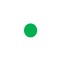	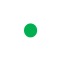	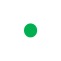	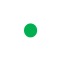				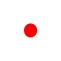
ESCAPE (2015)		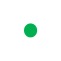	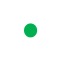	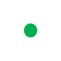	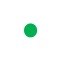	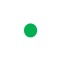	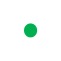			
EXTEND-IA (2015)		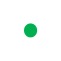	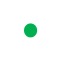	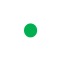	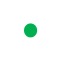	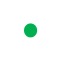	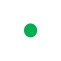			
SWIFT PRIME (2015)		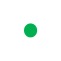	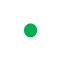	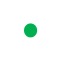	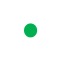	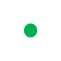	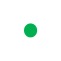			
REVASCAT (2015)		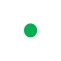	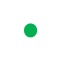	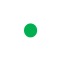	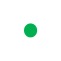	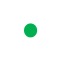	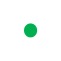			
THERAPY (2015)		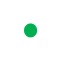	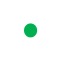							
THRACE (2015)			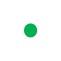							

EBT: endovascular bridging therapies; IAT: intra-arterial pharmacologic thrombolysis; ET: endovascular thrombectomy; IVT: intravenous rt-PA; RCT: randomized controlled trials.

**Table 2:** Basic characteristics and major conclusions of included meta-analysis.

Author (year)	Basic information: number, age	Quality of primary studies and meta-analysis	Outcome measure	Results of included meta-analysis	Major conclusions of included meta-analysis
ET + IVT versus IVT					

Singh et al. 2013	5 studies, 711/486 (1197); NA	Mostly good (Jadad scale) and 10/11 points	(1) mRS (3 months) (2) Mortality, sICH rate	No significant improvement in patients receiving ET compared with those receiving IVT; ET was found to have better outcomes in patients with severe stroke (NIHSS > 20)	ET is not superior to IV thrombolysis for acute ischemic strokes. ET may lead to a better outcome for patients with severe strokes (−)

Balami et al. 2015	8 studies, 1313/1110 (2423); 65 to 71 years	All good (Cochrane collaboration) and 9/11 points	(1) mRS (3 months) (2) Mortality, sICH rate	ET had a greater chance of a favorable primary outcome (OR 1.56, *p* < 0.00001). There was a tendency toward decreased mortality (OR 1.56, *p* = 0.12), and sICH was not increased (*p* = 0.88)	Clear evidence for improvement in functional independence with ET compared with IVT, suggesting that ET should be considered for AIS (+)

Elgendy et al. 2015	9 studies, 1363/1047 (2410); NA	All good and 10/11 points	(1) mRS (3 months) (2) Mortality, sICH rate (3) Recanalization	ET was associated with a higher incidence of achieving good functional outcome (43.7% versus 30.9%, *p* < 0.00001). ET was relevant to a trend toward reduction in the risk of all-cause mortality and improved recanalization (*p* < 0.05). The risk of in-hospital sICH was similar	ET could improve functional outcomes compared with IVT and was found to be relatively safe, with no excess in intracranial hemorrhage. There was a trend for reduction in all-cause mortality with ET (+)

Badhiwala et al. 2015	8 studies, 1313/1110 (2423); 64 to 72 years	All good (Cochrane collaboration) and 10/11 points	(1) mRS (3 months) (2) Function independence (3) Mortality, sICH rate (4) Recanalization	ET was benefit across mRS scores (*p* = 0.005); functional independence occurred more in the ET group (44.6% versus 31.8%); ET had higher rates of angiographic revascularization at 24 hours, but no significant difference in rates of sICH or all-cause mortality at 90 days (*p* > 0.05)	ET versus IVT was associated with improved functional outcomes and higher rates of angiographic revascularization, but no significant difference in symptomatic intracranial hemorrhage or all-cause mortality at 90 days (+)

Goyal et al. 2016	5 studies, 634/653 (1287); 57 to 77 years	All good and 10/11 points	(1) mRS (3 months) (2) Mortality, sICH rate (3) NIHSS	ET led to significantly reduced disability at 90 days compared with control; mortality at 90 days and risk of sICH did not differ between populations	ET is of benefit to most patients with AIS caused by occlusion of the proximal anterior circulation. These findings will have global implications on structuring systems of care to provide timely treatment to patients with AIS (+)

Bush et al. 2016	5 studies, 634/653 (1287); NA	All good (Cochrane collaboration) and 9/11 points	(1) mRS (3 months) (2) Mortality, sICH rate	Patients received ET experienced 2.22 times greater odds of better functional outcome compared to IVT (*p* < 0.0001); ET was not associated with mortality or symptomatic intracerebral hemorrhage (*p* > 0.05)	This meta-analysis showed superior functional outcomes in patients receiving ET Further, this analysis showed that AIS patients may receive enhanced functional benefit from earlier ET (+)

Campbell et al. 2016	4 studies, 401/386 (787); 67.8 ± 12.3 years	All good (Cochrane collaboration) and 10/11 points	(1) mRS (3 months) (2) Independent outcome (3) Mortality, sICH rate (4) Recanalization	The common odds ratio for mRS improvement was 2.7; successful revascularization occurred in 77% treated with ET; sICH rate and overall mortality did not differ (*p* > 0.05)	ET for large vessel ischemic stroke was safe and highly effective with substantially reduced disability (+)

IAT versus IVT					

Lee et al. 2010	5 studies, 224/171 (395); 61 to 68 years	Mostly good (Cochrane collaboration) and 10/11 points	(1) mRS (3 months) (2) NIHSS (3) Barthel Index (4) Recanalization (5) Mortality, sICH rate	IAT was associated with good and excellent outcome and could ameliorate NIHSS, Barthel Index, and increase recanalization. IAT was associated with increased sICH; no difference in mortality between groups	Formal meta-analysis suggests that IAT substantially increases recanalization rates and good clinical outcomes in AIS patients (+)

Fields et al. 2011	3 studies, 261/134 (395); 64 to 68 years	All good (Cochrane collaboration) and 8/11 points	(1) mRS (3 months) (2) NIHSS (3) Mortality, sICH rate	IAT were significantly more likely to have a good mRS and NIHSS. There was no effect on mortality at 90 days (20% versus 19%). The risk of SICH was significantly increased in the IAT groups (*p* = 0.12)	These meta-analyses support endovascular treatment of acute ischemic stroke due to MCA occlusion with IAT (+)

EBT versus IVT					

Fargen et al. 2015	6 studies, 1071/832 (1903); 18 to 85 years	All good (Cochrane collaboration) and 9/11 points	(1) mRS (3 months) (2) Mortality, sICH rate	EBT was associated with significantly improved outcomes compared with medical management (OR 1.67, *p* = 0.0001) for patients with LVO	This meta-analysis suggested that EBT produce superior clinical outcomes compared to medical management in AIS patients from LVO (+)

EBT: endovascular bridging therapies; IAT: intra-arterial pharmacologic thrombolysis; ET: endovascular thrombectomy; IVT: intravenous rt-PA; NA: not available; mRS: modified Rankin scale; sICH: symptomatic intracranial hemorrhage; OR: odds ratio; AIS: acute ischemic stroke; NIHSS: National Institutes of Health Stroke Scale Score; and LVO: large vessel occlusion.

**Table 3:** Efficacy outcomes of mRS score from the pooled data.

Author (year)	OR	95% CI	*p* value
ET + IVT versus IVT: mRS score (0–6)			
Badhiwala et al. 2015	1.56	1.14–2.13	0.0005
Goyal et al. 2016	2.26	1.67–3.06	0.0001
Bush et al. 2016	2.47	1.92–3.18	0.0001
Campbell et al. 2016	2.4	1.8–3.0	0.0001
IAT versus IVT: mRS score (0-1)			
Lee et al. 2010	2.14	1.31–3.51	0.003
Fields et al. 2011	1.97	1.15–3.35	0.01
IAT versus IVT: mRS score (0–2)			
Lee et al. 2010	2.05	1.33–3.14	0.001
Fields et al. 2011	1.86	1.15–2.99	0.01
EBT versus IVT: Fargen et al. 2015			
mRS score (0-1)	1.22	0.97–1.53	0.09
mRS score (0–2)	1.27	1.04–1.54	0.018
mRS score (0–3)	1.25	1.04–1.51	0.019

EBT: endovascular bridging therapies; ET: endovascular thrombectomy; IAT: intra-arterial pharmacologic thrombolysis; IVT: intravenous rt-PA; mRS: modified Rankin scale; OR: odds ratio; and 95% CI: 95% confidence interval.

**Table 4:** Efficacy of secondary outcomes from the pooled data.

	ET + IVT versus IVT						IAT versus IVT		EBT versus IVT
Primary studies of meta-analysis	Singh et al. 2013	Balami et al. 2015	Elgendy et al. 2015	Badhiwala et al. 2015	Goyal et al. 2016	Campbell et al. 2016	Lee et al. 2010	Fields et al. 2011	Fargen et al. 2015
*Mortality*									
N1/T1	127/707	206/1312	15.9%	218/1312	97/633	48/401	46/224	40/201	203/1071
N2/T2	84/490	194/1106	17.9%	201/1106	122/646	63/386	41/171	24/130	156/832
OR 95% CI	0.98 (0.76–1.25)	0.84 (0.64–1.05)	0.82 (0.67–1.02)	0.87 (0.68–1.12)	0.77 (0.54–1.10)	0.69 (0.43–1.1)	0.83 (0.48–1.39)	0.84 (0.47–1.52)	0.96 (0.76–1.22)
*p* value	>0.05	0.12	0.08	0.27	0.16	0.12	0.46	0.57	0.73
*sICH rate*									
N1/T1	42/707	66/1312	5.1%	70/1312	28/633	10/401	20/224	20/201	
N2/T2	30/490	53/1106	5.0%	53/1106	28/646	11/386	4/171	3/130	
OR 95% CI	0.99 (0.62–1.58)	1.03 (0.71–1.49)	1.02 (0.69–1.52)	1.12 (0.77–1.63)	1.07 (0.62–1.83)	0.87 (0.36–2.1)	2.87 (1.21–6.83)	4.58 (1.31–15.97)	
*p* value	>0.05	0.88	0.92	0.56	0.8	0.76	0.02	0.02	
*Revascularization*									
OR 95% CI			3.09 (2.46–3.89)	6.49 (4.79–8.79)					
*p* value			0.0001	<0.01					

EBT: endovascular bridging therapies; ET: endovascular thrombectomy; IAT: intra-arterial pharmacologic thrombolysis; IVT: intravenous rt-PA; mRS: modified Rankin scale; OR: odds ratio; and 95% CI: 95% confidence interval.

## Supplementary Materials

Supplementary Materials 2009 PRISMA Checklist for meta-analysis.
